# Development of chia gum/alginate-polymer support for horseradish peroxidase immobilization and its application in phenolic removal

**DOI:** 10.1038/s41598-024-51566-x

**Published:** 2024-01-16

**Authors:** Saleh A. Mohamed, Alshaimaa M. Elsayed, Hala A. Salah, Amal Z. Barakat, Roqaya I. Bassuiny, Heidi M. Abdel-Mageed, Azza M. Abdel-Aty

**Affiliations:** Molecular Biology Department, National Research Centre, Dokki, Cairo, Egypt

**Keywords:** Biochemistry, Biological techniques

## Abstract

Chia gum’s molecular structure with distinctive properties as well as the alginate-based hydrogel’s three-dimensionally cross-linked structure can provide a potent matrix for immobilization of enzyme. Herein, chia gum (CG)/alginate (A)-polymeric complex was synthesized and employed as a support material for the immobilization of horseradish peroxidase (HRP). HRP was successfully immobilized on the developed ACG-polymeric support, and the highest immobilization recovery (75%) was observed at 1.0% CG and 2% A, pH 7.0, and 50 units of the enzyme. The structure, morphology, and thermal properties of the prepared ACG-HRP were demonstrated using Fourier Transform Infrared (FTIR), Scanning Electron Microscope, and Thermogravimetric (TGA) analyses. ACG-HRP showed a good reusability (60%) over ten reuses. The immobilized ACG-HRP displayed an acidic pH optimum (6.0), a higher temperature optimum (50 °C), and improved thermal stability (30–50 °C) compared to the soluble HRP at pH 7.0, 40 °C and (30–40 °C), respectively. ACG-HRP has a lower affinity for hydrogen peroxide (H_2_O_2_) and guaiacol and a higher oxidizing affinity for a number of phenolic substrates. The ACG-HRP demonstrated greater resistance to heavy metals, isopropanol, urea, Triton X-100, and urea, as well as improved efficiency for eliminating phenol and *p*-chlorophenol. The developed ACG-polymeric support provided improved enzyme properties, allowed the reuse of the immobilized HRP in 10 cycles, and made it promising for several biotechnological applications.

## Introduction

Plant peroxidase has been used for removing phenolic compounds and dyes from wastewater^1–5^. However, soluble peroxidases had several limitations, including instability at different pHs, temperatures, solvents, surfactants, non-reusability, and partial degradation of pollutants due to enzymes' denaturation^6^. They couldn’t also be utilized in continuous reactors^7^. To overcome such restrictions, peroxidases should be immobilized on solid supports for long-term utilization on a large scale^8,9^. Entrapment is a better choice among various techniques employed for immobilizing enzymes. Entrapment preserves the enzyme's original structure and generates a non-reactive aqueous microenvironment inside the matrix^10^. Earlier, matrices including gelatin, agar–agar, chitosan, agarose, polyacrylamide, carrageenan, sugar ester, alginate, and pectin were used to entrap enzymes^11,12^. However, some disadvantages are often associated with these matrices, such as leakage of entrapped enzymes from the support during its application and low mechanical strength^13^. To resolve these disadvantages, these polymeric matrices have been coupled with other polysaccharides like plant gums with distinctive/unique properties including high emulsification, high solubility, low cost, good volatile retention, and negatively charged characteristics.

Chia seed gum that is present in the seed coat is readily apparent and extractable when exposed to water. Chia-gum is an anionic heteropolysaccharide with a molecular weight ranging from 800 to 2000 kDa^14^. Chia gum is composed of β-d-xylopyranosyl, α-d-glucopyranosyl, and 4-O-methyl-α-d-glucopyranosyluronic acid units in the ratio 2:1:1 in a repeating unit producing chains. It exhibited a greater capacity for retaining, absorbing, and binding water^15^. Due to its hydrophilic properties, it can entrap a large amount of water between its chains and prevent water loss from entrapping support, thus obstructing the leaching of the enzyme. In addition, gums are negatively charged, while proteins are positively charged. These polymers interact because of their opposite charges, resulting in complex coacervation. Many applications for coacervation, include delivering bioactive compounds in microcapsules, stabilizing emulsions, and encapsulating polyunsaturated oils and other bioactive food ingredients^16–18^.

Alginate is generally employed in entrapping many enzymes, such as catalase, laccase, amylase and horseradish peroxidase^7,19–21^. Sodium alginate has a specific advantage when calcium ions are present, producing a physically cross-linked hydrogel. Therefore, in the current study, the chia gum (CG) and alginate hydrogels (A) were mixed/combined to create polymeric support for entrapping the horseradish peroxidase (HRP). Several parameters/conditions were optimized to maximize enzyme entrapment within the prepared polymeric support. A comparative stability study was done for soluble HRP and ACG-HRP against various physical and chemical parameters. The removal of phenolic compounds was also evaluated for soluble HRP and ACG-HRP.

## Materials and methods

Horseradish peroxidase (HRP) was previously purified by Mohamed et al.^22^. The purified HRP belongs to class III (plant-secreted peroxidases). Chia seed was purchased from local market Cairo, Egypt.

### Chia gum preparation

Chia seeds were soaked in distilled water at a ratio of 1:30 for 1 h at 40 °C. The obtained gum was separated by filtration and three volumes of ethanol were added to one volume of gum. The precipitate was collected, dried, and ground.

### Peroxidase activity determination

The peroxidase activity was measured in the presence of 20 mM sodium acetate buffer, pH 5.5, 8 mM H_2_O_2,_ 40 mM guaiacol, and HRP enzyme according to Miranda et al.^23^ with some modifications. The change in absorbance (A_470_) 1.0/min is considered one unit.

### Immobilization process

**Scheme 1:**
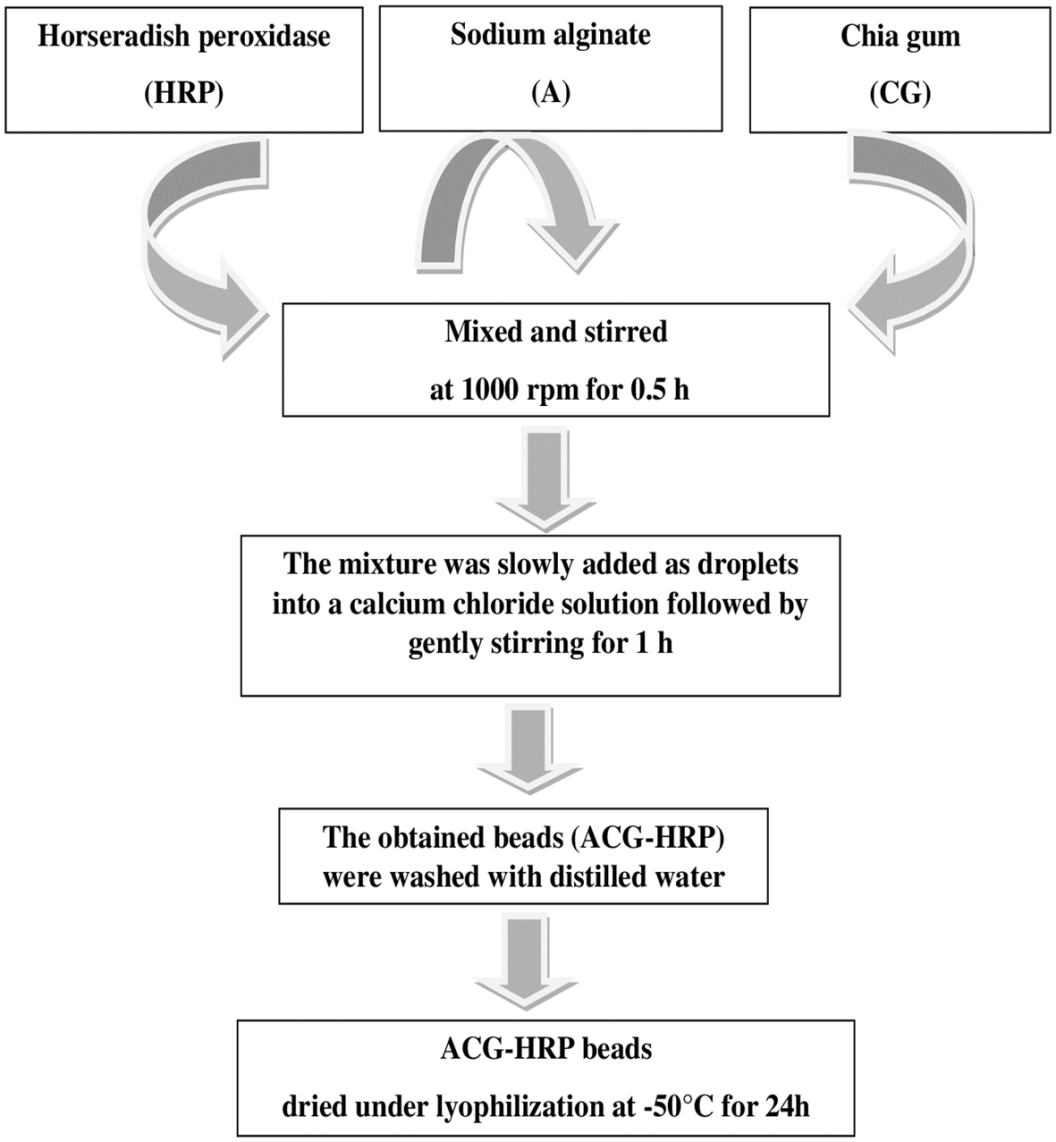
The immobilization process steps for preparation of the ACG-HRP beads.

HRP (50 U) was mixed with 5 ml sodium alginate (A) (2%, w/v) and 2.5 ml chia gum (CG) (1%, w/v) with stirring at 1000 rpm for 0.5 h. The resulting mixture was slowly extruded as droplets into 7.5 ml calcium chloride solution (3.0%, w/v) and further gently stirred for 1 h. The obtained beads (ACG-HRP) were washed with distilled water and dried under lyophilization at − 50 °C and for one day and stored at 4 °C for further use. Scheme 1 summarizes the steps of the immobilization process. The immobilization efficiency % was calculated from the following equation.

$$Immobilization\,\,efficiency\,(\% ) = \frac{Activity\,of\,immobilized\,enzyme}{{Intial\,activity\,of\,soluble\,enzyme}} \times 100$$

### Surface morphology

The surface morphology study of supports (ACG) and the immobilized enzyme (ACG-HRP) was analyzed using Holland Field Emission Scanning Electron Microscope (FE-SEM, QUANTA FEG250) with an accelerating voltage of 20 kV.

### Fourier transform infrared analysis (FTIR-analysis)

The FTIR Spectrometer (Bruker ALPHA-FTIR-Spectrometer) was employed to take spectra of ACG and ACG-HRP. Platinum-attenuated reflection was used at a wave range of 400–4000 cm^1^.

### Thermal analysis

The thermal properties of ACG and ACG-HRP were evaluated by non-isothermal thermogravimetric (TGA) and differential thermogravimetric (DTG) analyses. Sample runs were performed at a constant heating rate of 10 °C/min from 40 to 800 °C.

### Biochemical characterization

The reusability of the ACG-HRP was determined by the removal of the enzyme support from the reaction mixture and washed with 20 mM sodium acetate buffer at pH 5.5. And then it was reused one more time in the same reaction mixture. The optimal pH of the ACG-HRP and soluble HRP was measured at different pH values (4.0–9.0). The optimal temperature of the ACG-HRP and soluble-HRP was measured under different temperatures (20–80 °C) using standard assay as described above. In the temperature-stability assay, either the ACG-HRP or the soluble-HRP was incubated for 1 h at different temperatures (30–80 °C) preceding the substrate addition. And the remaining activity was measured as described above. The Km values were calculated using H_2_O_2_ or guaiacol at different concentrations. The effect of some metal ions and some denaturation compounds on the activity of both the ACG-HRP and soluble-HRP was also measured by preincubation of the enzyme for 30 min with these compounds or metal ions preceding the substrate addition. And the remaining activity was measured as described above.

### Removal of phenolic compounds

The removal of phenol or *p*-chlorophenol was determined according to the method of Bhunia et al.^24^ The reaction mixture includes 2 mM phenol, 4 mM H_2_O_2_ and soluble HRP or ACG-HRP. The reaction mixture was taken at 1 h intervals and transferred to 2.0 mM *p*-aminoantipyrine and 6 mM potassium ferricyanide. The absorbance was measured at 510 nm.

All experimental procedures were carried out in compliance with relevant guidelines.

## Results and discussion

### Immobilization of HRP on ACG

Different percentages of the CG (0.5–2.0%) and different pH’s (5.0–8.0) were tested for immobilization of HRP (Table 1). 50 units of HRP and 2% of A were employed in the experiment. The maximum immobilization efficiency (75%) was obtained at 1.0% CG and pH 7.0. The minimum immobilization efficiency (33%) was observed at the highest percentage of the GC (2.0%) and pH 8.0. This reduction in the immobilization efficiency may be due to a high load of gum which led to a change in the enzyme structure and reduced its activity. This inverse relationship was previously highlighted. Among previous studies, immobilized-HRP showed different immobilization capacities, for instance, the immobilized-HRP on lysine-functionalized gum Arabic-coated Fe_3_O_4_ nanoparticles recorded immobilization efficiency of 60%^25^.

**Table 1:** Effect of different concentrations of chia gum and pHs on the immobilization efficiency of HRP.

Gum concentration %	Immobilization efficiency %		
	pH 5.0	pH 7.0	pH 8.0
0.5	50 ± 2.2	58 ± 2.4	40 ± 3.2
1.0	63 ± 3.1	75 ± 3.0	50 ± 3.5
2.0	42 ± 1.9	50 ± 2.3	33 ± 2.1

**Figure 1:**
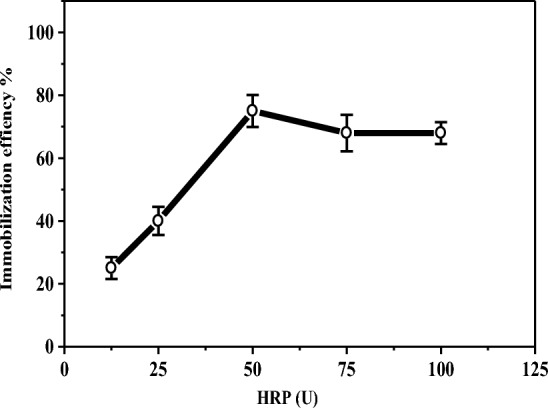
Effect of HRP different concentrations (12.5-100U) on the immobilization efficiency, the enzyme activity examined under standard assay conditions.

Figure 1 shows the effect of various concentrations of HRP (12.5–100 units) on the immobilization efficiency % under the best immobilization conditions (pH 7.0 and 1.0% CG) as obtained above. The maximum immobilization efficiency (75%) was recorded at 50 units. The overload of enzyme concentration (100 units) showed a slight decline in the immobilization efficiency (68%). This decline may be due to over-accumulation of the enzyme on the polymer surface leading to a reduction of the substrate diffusion^3^.

### Surface-morphology characterization

**Figure 2:**
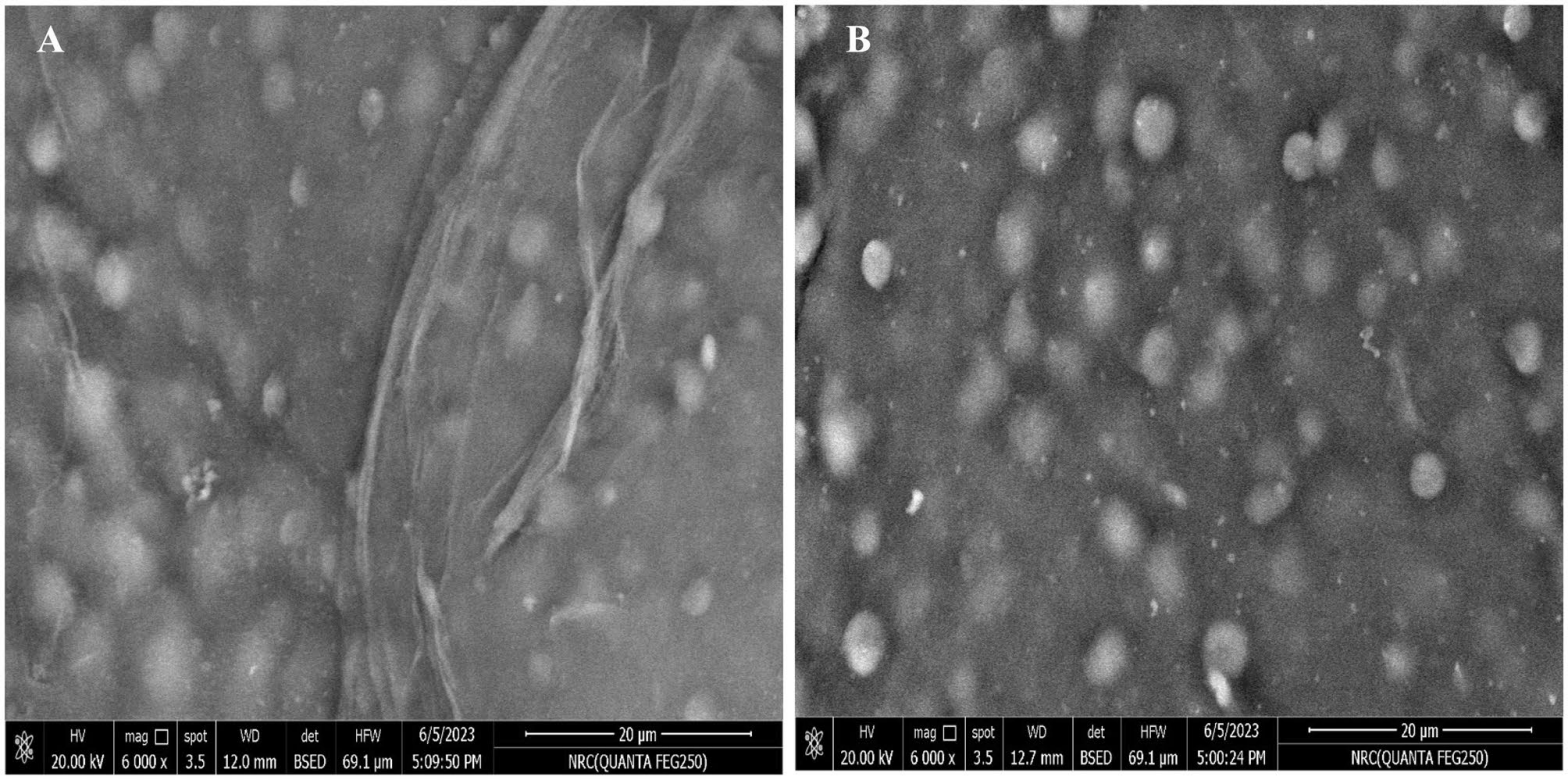
SEM micro-images of the prepared ACG-polymeric support (**A**) and immobilized ACG-HRP (**B**).

SEM micro-images were used to study the surface morphology of the ACG-polymeric support and immobilized ACG-HRP. Alginate beads often have a structure that is highly porous, as previously seen in SEM images^26^. Figure 2A indicates that compact pores were found on the ACG beads/ridges’ smooth surface. This might be due to the covering of alginate pores by chia gum. Additionally, the HRP displayed small particles with irregular shapes on the surface of ACG beads/ridges demonstrating the covering/entrapment of HRP on the ACG beads (Fig. 2B). Such covering and surface morphology changes of the immobilized enzyme on the alginate beads were also previously observed^26–28^.

### FTIR-analysis

**Figure 3:**
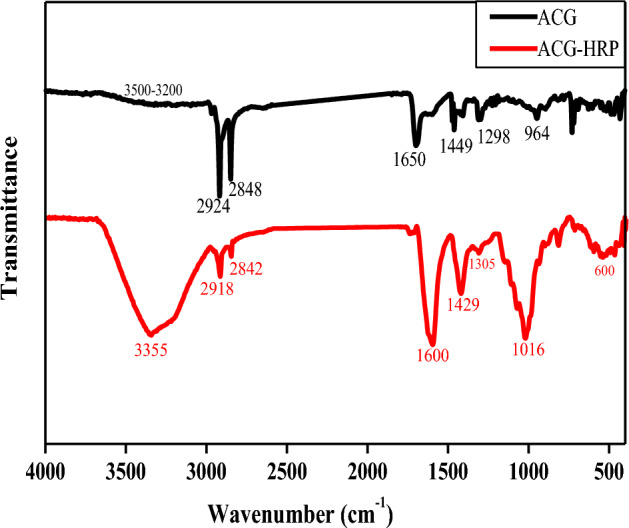
FTIR spectra of the prepared ACG-polymeric support and immobilized ACG-HRP.

The FTIR spectrum of ACG-polymeric support is presented in Fig. 3. The characteristic vibrations between 3500 and 3100 cm^−1^ represent the hydroxyl (OH) stretching peaks that provided the basic structure of polysaccharides. Symmetric and asymmetric stretching bands of C–H_2_ at 2924 and 2848 cm^−1^, are commonly generated from sugars and carbohydrates, respectively. Gums typically contain uronic acids, usually giving the macromolecule an anionic property^29^. The peaks at 1650 and 1449 cm^−1^ appear to be caused by the asymmetric and symmetric vibrations of the carboxyl group (COO–) of uronic acids. Additionally, the band at 1298 cm^−1^ is attributed to the C–O stretching vibration, and the sharp peak at 964 cm^−1^ is assigned to C–C and to C–O–C vibrations. These identified peaks were commonly found in both gums, mucilage, and calcium alginate structures^18,30^ suggesting that this structure derived from the interaction between A and CG. On the other hand, the immobilized ACG-HRP beads-spectrum displayed significant difference bands compared to the ACG spectrum (Fig. 3). The absorption region of stretching vibration for the hydroxyl band (OH) in ACG-HRP appeared wider than in ACG, indicating the overlap of OH and amide A band (N–H stretching) typically found in HRP at 3355 cm^−1^. In addition, the amide groups of the HRP including amide I (C=O stretching), amide II (N–H deformation), and amide III (C–N stretching and N–H bending) are also absorbed/appeared at 1600, 1429, 1016 cm^−1^, respectively, and C–H bond bending or deformation demonstrated at 600 cm^1^. The appearance of these groups indicates an amidation reaction and confirms the immobilization of HRP on the ACG support^31–33^. Further, the bands/peaks at 2924, 2848, 1650, 1449, 1298, and 964 cm^−1^ are shifted to 2918, 2842, 1600, 1429, 1305, and 1016 cm^−1^, respectively. Overall, shifting, overlapping, intensity variations, and peroxidase functional groups identified in the ACG-HRP bands/structure suggest a potential interaction between the ACG support and the peroxidase enzyme following immobilization. These findings were previously described^34^.

### Thermal characterization

**Figure 4:**
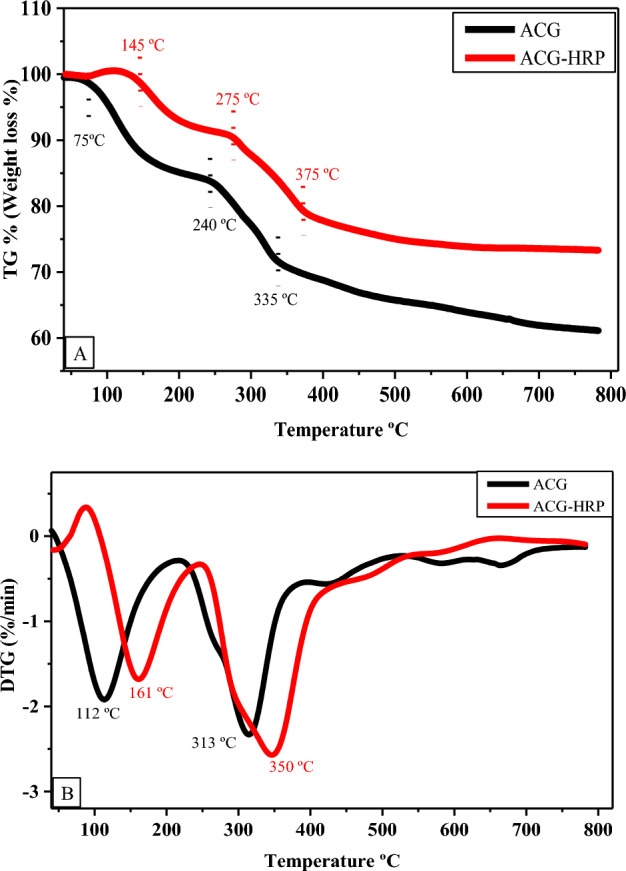
(**A**) TGA and (**B**) DTG curves of the ACG-support and immobilized ACG-HRP.

The prepared ACG-HRP and the ACG-complex thermal stability were compared using TGA and DTG examinations. According to TGA-Fig. 4A, the early thermal degradation/dehydration of the ACG complex occurred between 75 and 240 °C and resulted in a mass loss of 3–17%. While the initial thermal degradation of the ACG-HRP occurred between 145 and 275 °C and caused a lesser mass loss of 2–9%. The initial mass loss is caused by removing the water/moisture entrapped in the molecular structure^35^. Additionally, the second mass decomposition was found around 240–335 °C for ACG-complex and 275–375 °C for ACG-HRP, and they retained 71 and 80% of their weights, respectively. Finally, at 800 °C, the ACG complex kept 60% of its weight while ACG-HRP retained 75% of its weight. The steady weight loss of immobilized ACG-HRP confirms enhanced thermal stability due to its highly organized structure. As demonstrated in DTG-Fig. 4B, the maximum decomposition rate of ACG-support and immobilized ACG-HRP was found at two-step decomposition temperatures of 112, 313 °C, and 161, 350 °C, with decomposition rates of − 1.9, − 2.3 and − 1.6, − 2.5, respectively. The observation that immobilized ACG-HRP exhibits higher initial and final decomposition temperatures than ACG-support and retained a higher weight demonstrates that ACG-HRP's thermal stability has increased/improved as a result of HRP interaction with ACG-support. Additionally, it is evident that the ACG-HRP structure maintained a large HRP-enzyme weight fraction^36^.

### Biochemical characterization

**Figure 5:**
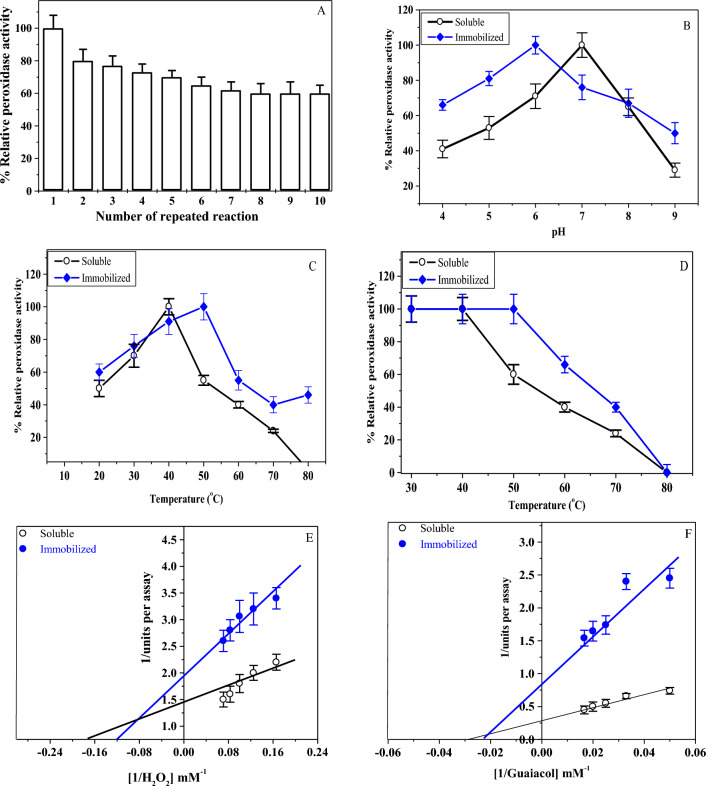
(**A**) Number of reuses of the immobilized ACG-HRP and retained activity. (**B**) pH-optimum for soluble and immobilized enzyme. (**C**) Temperature-optimum and (**D**) Thermal-stability using different temperatures of the ACG-HRP compared to the soluble HRP-enzyme. Lineweaver–Burk plots of soluble and immobilized ACG-HRP reaction velocities using different concentrations of H_2_O_2_ (**E**) and guaiacol (**F**).

One of the most economical advantages of the immobilized enzyme over the free enzyme is that it can be reused several times. In the current study, the reusability of the ACG-HRP was evaluated during 10 reuses as seen in Fig. 5A. The results revealed that the ACG-HRP retained 60% of its original activity after 10 reuses suggesting that the ACG-HRP possessed high stability and can be re-used several times. As for the reduction in the enzyme activity over multiple reuses, as expected, may be due to the separation and breakage of the support^37,38^. Similarly, the immobilized HRP on the tyramine-alginate, lysine-functionalized gum Arabic-coated Fe_3_O_4_ nanoparticles and polyvinyl alcohol-alginate retained 61%, 60% and 64% of its initial activity after 4, 8 and 10 consecutive cycles of use^25,39,40^.

The pH-optimum, temperature-optimum, and thermal stability of the immobilized ACG-HRP were observed at pH 6.0, 50 °C and (30–50 °C) compared to the soluble-HRP at pH 7.0, 40 °C and (30–40 °C), respectively as shown in Fig. 5B, C and D. These observations indicate that ACG-HRP could resist the effects of the alkaline medium and be more thermally stable than the free enzyme. Similarly, peroxidase-ZnO/SnO_2_/alginate nanocomposite and HRP-Ca alginate-starch beads were optimally active at pH 5, 50 °C and pH 6, 50 °C, respectively^41,42^. However, the optimum pH and thermal stability shifted from 7.0 to 8.0 and 60 to 70 °C after immobilization of HRP on the tyramine-alginate, lysine-functionalized gum Arabic-coated Fe_3_O_4_ nanoparticles, respectively ^25^.

Further, the Km values of the immobilized ACG-HRP and soluble-HRP were found to be 8.33 and 5.88 mM for H_2_O_2_ and 45 and 35.7 mM for guaiacol, respectively (Fig. 5E,F), suggesting that the immobilized enzyme has lower affinity toward substrates. Similar to our findings, several studies reported that the immobilized HRP had higher Km values toward these substrates than the free-form. The Km of HRP-Ca alginate-starch beads and free HRP were 62.93 and 14.53 mM for H_2_O_2_ and 136.69 and 7.83 mM for guaiacol, respectively^42^. On the contrary, Yapaoz and Attar showed that the no change of Km values for entrapped HRP in alginate and free enzyme^43^. Such shifts/changes in the biochemical and kinetic properties of the immobilized HRP were induced as a result of the immobilization process, where the enzyme structure is altered after conjugation with the support, which impacts the catalytic site and the substrate diffusion site^44,45^.

Table 2 shows the effect of some metal ions on the enzyme activity either in its immobilized form or soluble form. All the investigated metals showed a low inhibitory effect on the ACG-HRP compared to soluble HRP. Zn^2+^, Mg^2+^, and Cu^2+^ showed no effect on ACG-HRP. Further, Hg^2+^ has a higher inhibitory effect on the soluble enzyme. These results indicate that the immobilization process improved the enzyme structure to resist the heavy-metals action and the ACG-HRP can be used for wastewater treatment which contains high levels of heavy metals. Similarly, at 4.0 mM HgCl_2_ the soluble pointed gourd peroxidase lost 67.6% of initial activity whereas peroxidase–concanavalin A complex on calcium alginate pectin gel retained 65%^46^.

**Table 2:** Effect of some metal ions at concentration of 10 mM on the soluble HRP and ACG-HRP.

Metals	Relative peroxidase activity %	
	Soluble-HRP	ACG-HRP
None	100 ± 4.3^a^	100 ± 3.1^a^
Ni^2+^	21 ± 6.0^b^	50 ± 18.5^b^
Zn^2+^	29 ± 5.1^b^	100 ± 20.2^b^
Mg^2+^	60 ± 4.2^b^	98 ± 11.5^b^
Ca^2+^	58 ± 5.6^a^	67 ± 10.5^b^
Al^2+^	42 ± 3.3^b^	74 ± 5.2^a^
Cu^2+^	59 ± 4.3^b^	100 ± 6.2^a^
Hg^2+^	11 ± 3.1^b^	50 ± 5.8^a^

Values with different superscripts (a, b) were significantly different at (*P* < 0.01).

The immobilized ACG-HRP showed higher oxidizing affinity toward the tested substrates (guaiacol, *o*-dianisidine, *o*–phenylenediamine, pyrogallol, and *p*-aminoantipyrine) than soluble-HRP as seen in Table 3, suggesting that the enzyme immobilization process improved/altered the structure of substrate-binding site of the enzyme. Similarly, the HRP immobilized on the CMPS possessed a higher affinity to these substrates^3^.

**Table 3:** Substrate specificity of the soluble HRP and ACG-HRP.

Substrate	Relative peroxidase activity %	
	Soluble-HRP	ACG-HRP
Guaiacol	100 ± 4.1^a^	100 ± 8.0^a^
*o*-Dianisidine	32 ± 6.4^b^	85 ± 19.2^b^
*o*-Phenylenediamine	60 ± 5.0^b^	79 ± 10.2^b^
Pyrogallol	12 ± 2.8^b^	69 ± 11.7^b^
*p*-Aminoantipyrine	10 ± 3.8^b^	41 ± 8.2^b^

Values with different superscripts (a, b) were significantly different at (*P* < 0.01).

Table 4 depicts the influence of some compounds on the activity of both immobilized and soluble-HRP. At 2.0 M urea, the immobilized ACG-HRP retained 49% of its original activity while the soluble-HRP retained 38%, suggesting that the immobilization process altered the HRP structure to resist urea action which causes protein-unfolding and declines the enzyme activity. Similarly, peroxidase–concanavalin A complex on calcium alginate pectin gel showed higher resistance against urea than the soluble HRP with 40% and 80% loss of its activity, respectively^46^. At 10% Triton X-100, the activity of the ACG-HRP decreased to reach 60% of its activity, while the soluble-HRP retained only 18%. The resistance of the immobilized ACG-HRP against Triton X-100 is a very important result, where the immobilized ACG-HRP can tolerate the detergent effect during wastewater treatment. The soluble pointed gourd peroxidase retained 22.9% of initial activity at 6% Triton X-100 compared with 42.8% for peroxidase–concanavalin A complex on calcium alginate pectin gel^46^.

**Table 4:** Effect of some inhibitors on the soluble HRP and ACG-HRP.

Substrate	Concentration	Relative peroxidase activity %	
		Soluble-HRP	ACG-HRP
None	–	100 ± 5.3^a^	100 ± 4.2^a^
Urea	2 M	38 ± 1.8^b^	49 ± 2.2^b^
Triton X-100	10%	18 ± 2.0^b^	60 ± 1.5^b^
Isopropanol	10%	56 ± 1.7^b^	73 ± 4.7^b^

Values with different superscripts (a, b) were significantly different at (*P* < 0.01).

Organic solvents have a negative influence on the peroxidase activity during wastewater processing. Isopropanol is one of the most important organic solvents that inhibit peroxidase activity. The ACG-HRP retained 73% of its activity under 10% isopropanol treatment, while the soluble-HRP retained 56% of its activity, suggesting that the prepared ACG-HRP can efficiently resist the organic solvents already found in wastewater. Yapaoz and Attar showed that the entrapped HRP in alginate had higher residual activity in organic solvents compared to the free enzyme^43^.

**Figure 6:**
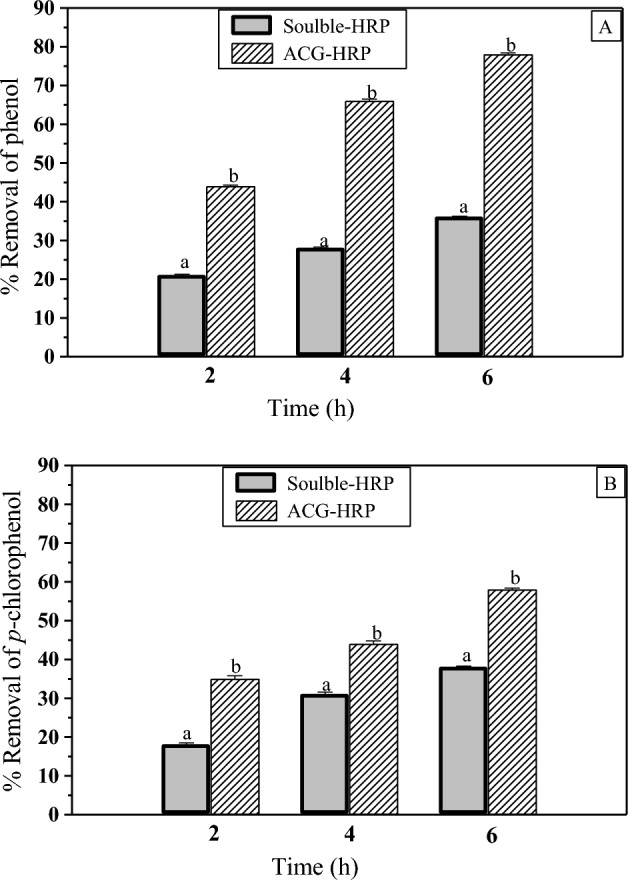
Effect of soluble-HRP and CGA-HRP on phenol (**A**) and p-chlorophenol (**B**) removal. Values are presented as means ± SE (n = 3). Values with different superscripts (**a**, **b**) were significantly different at (*P* < 0.01).

Phenolic compounds are considered one of the most hazardous pollutants in industrial wastewater. Figure 6 shows the effect of incubation time on the removal of phenol and *p*-chlorophenol using soluble and ACG-HRP. The results showed that ACG-HRP possessed a higher effectiveness for the removal of phenol and *p*-chlorophenol (78 and 58%, respectively) compared to soluble-HRP (36 and 38%, respectively) during 6 h of incubation. Study on time course of phenol removal for both HRP-alginate and soluble enzyme showed that immobilized enzyme had higher efficiency (60% removal of phenol) in comparison with soluble enzyme (50% removal of phenol)^47^.

## Conclusion

In this study the alginate and chia gum polymeric support (ACG) was developed for the immobilization of HRP. The immobilized ACG-HRP provided a high yield of immobilization recovery and good reusability. The effective binding of HRP on the surface of the ACG-support was evaluated and confirmed by SEM, FTIR, and TGA analyses. The immobilization process highly improved the enzyme stability against many denaturation factors such as pH, heat, heavy metals, detergents, organic solvents, and urea besides its oxidation activity toward several phenolic substrates. Thus, the ACG hydrogel is a good candidate for immobilization of HRP, increases its stability, and can be used in the removal of phenolic compounds.

### Future challenges and prospects

The use of plant gums in immobilization of enzymes compared to alginate is considered limited. The present study showed the vital role of chia gum for immobilization of HRP. Several studies extracted gums from plants and studied their chemical and physical properties. The extraction of these gums is very simple and low cost compared with synthetic polymers. As a consequence, employing these gums in enzyme entrapment needs more studies.

## Data Availability

The datasets generated during and/or analyzed during the current study are available from the corresponding author on reasonable request.
